# Obesogenic and diabetic effects of CD44 in mice are sexually dimorphic and dependent on genetic background

**DOI:** 10.1186/s13293-022-00426-2

**Published:** 2022-04-11

**Authors:** Melissa VerHague, Jody Albright, Keri Barron, Myungsuk Kim, Brian J. Bennett

**Affiliations:** 1 Nutrition Research Institute, University of North Carolina Kannapolis, Kannapolis, NC 28081 USA; 2 Obesity and Metabolism Research Unit USDA, ARS Western Human Nutrition Research Center, 430 W Health Sciences Drive, Davis, CA 95616 USA; 3 Department of Nutrition, University of California, Davis, 95616 USA; 4 Korea Institute of Science and Technology (KIST), Gangneung, Gangwon-do Republic of Korea

**Keywords:** CD44, Obesity

## Abstract

**Introduction:**

CD44 is a candidate gene for obesity and diabetes development and may be a critical mediator of a systemic inflammation associated with obesity and diabetes.

**Methods:**

We investigated the relationship of CD44 with obesity in CD44-deficient mice challenged with a high-fat diet.

**Results:**

In mice fed a diet high in fat, cholesterol, and sucrose for 12 weeks fat mass accumulation was reduced in CD44-deficient mice bred onto both a C57BL/6J and the naturally TLR deficient C3H/HeJ background. Reduced fat mass could not be attributed to lower food intake or an increase in energy expenditure as measured by indirect calorimetry. However, we observed a 40–60% lower mRNA expression of the inflammation markers, F4/80, CD11b, TNF-α, and CD14, in adipose tissue of CD44-deficient mice on the C57BL/6J background but not the C3H/HeJ background, perhaps indicating that alternative factors may be affecting adiposity in this model. Measures of hepatic steatosis and insulin sensitivity were improved in CD44-deficient mice on a C57BL/6J but not in the C3H/HeJ mice. These results were highly sexually dimorphic as there were no detectable effects of CD44 inactivation in female mice on a C57BL/6 J or C3H/HeJ background.

**Conclusion:**

CD44 was associated with adiposity, liver fat, and glucose in male mice. However, the effects of CD44 on obesity may be independent of TLR4 signaling.

**Supplementary Information:**

The online version contains supplementary material available at 10.1186/s13293-022-00426-2.

## Introduction

Obesity is now well described as an inflammatory disorder, and cells of the innate immune system are critical for mediating adipose tissue development [1, 2] and systemic disease, including diabetes and insulin resistance [3]. One inflammatory protein associated with both obesity and the metabolic consequences of obesity is CD44, a cell-adhesion surface protein ubiquitously expressed and a primary ligand of hyaluronic acid. CD44 expression is associated with altered immune function in mice and has been reported to affect atherosclerosis and diabetes [4]. For example, a gene expression-based GWAS in both mice and humans found that CD44 likely plays a critical role in the development of adipose tissue inflammation and insulin resistance [5], and obese mice have increased adipose expression of CD44 [6, 7]. Furthermore, treatment of diabetic mice with an anti-CD44 monoclonal antibody reduced adipose inflammation and improved insulin sensitivity [8]. Observational studies in humans have found increased CD44 expression in insulin-resistant subjects [9] and reduced CD44 expression in patients responding strongly to weight loss therapy [10], indicating that circulating CD44 is a marker of susceptibility to these diseases and perhaps a therapeutic target.

Genetic polymorphisms have been clearly demonstrated to affect susceptibility to obesity as dozens of loci and SNPs have been identified in overweight humans [11–13] and mice [14–16]. For example, natural genetic variation between C57BL/6J and C3H/HeJ affects susceptibility to obesity and insulin resistance [17, 18]. In order to better understand the underlying genetic factors for these strain differences, recent studies from our laboratory used these two inbred mouse strains and identified CD44 as a critical mediator of a coexpression network associated with inflammatory response and metabolic traits in mice [19]. Furthermore, we found that perturbation of CD44 dramatically affects the network and has possible pleiotropic effects on metabolism.

Our current study investigates our hypothesis that CD44 is a critical mediator of obesity and metabolic syndrome. We measured the effects of CD44 deficiency in both C57BL/6J and C3H/HeJ inbred mouse strains challenged with a high fat, high sucrose, obesity-inducing diet. We chose C3H/HeJ mice as they are known to have a perturbed inflammatory response due to a functional mutation in the TLR4 gene [20] and are resistant to diet-induced obesity [21]. We report the effects of CD44 inactivation on adiposity, liver fat accumulation, and glucose tolerance in both male and female mice on C57BL/6J and C3H/HeJ genetic backgrounds to refine the mechanism(s) by which CD44 affects susceptibility to obesity and diabetes.

## Methods

### Animals

All procedures were approved by the IACUC at the University of North Carolina (UNC) Chapel Hill. Three mouse strains were purchased from Jackson Labs: C57BL/6J mice (B6) (stock #000664), CD44 knockout mice on a C57BL/6J background (CD44.B6) (stock #005085), and C3H/HeJ mice (C3H) (stock #000659). The fourth group of mice was created by selectively back-crossing CD44.B6 mice with C3H mice for at least 10 generations resulting in a genotyping-confirmed > 99% C3H background to result in CD44 knockout on a C3H/HeJ background (CD44.C3H). For all four groups, 8-week-old mice were fed a high fat (33% kcal fat from cocoa butter), high sucrose (48% kcal carbohydrates, with 80% carbs from sucrose), high cholesterol (1.25%) diet for 12 weeks (cat # D10042101, Research Diets, Inc., NJ, USA). Following a 4 h fast, mice were euthanized via isoflurane inhalation. Blood was collected via retro-orbital bleed into a BD Microtainer tube with K_2_EDTA (cat # 365974, Becton, Dickinson and Co., NJ, US), and organs were flushed with saline. Gonadal fat, inguinal fat, liver, cecum, and left kidney were harvested and snap-frozen in liquid N_2_.

### Body composition and metabolic analysis

For body composition analysis, lean and fat mass were measured every two weeks, beginning at the start of diet, using EchoMRI™. For metabolic analyses, one week before necropsy mice were individually housed in metabolic chambers (TSE Calorimetry Systems, Chesterfield, MO, USA) for 3 days with free access to food and water. This system is designed to simultaneously and continuously monitor food and water consumption, O_2_ consumption, CO_2_ production, and ambulatory movement using a photobeam break system. Data were collected every 3 min for 48 h, with the first 24 h considered an acclimation period and excluded from analyses. Oxygen consumption was calculated per gram of lean body weight.

### Glucose tolerance test and analysis of plasma lipids

Two weeks prior to necropsy, mice were subjected to a glucose tolerance test. After an 8-h fast, mice were administered either 1 g/kg body weight or 2 g/kg body weight of glucose via intraperitoneal injection. Blood glucose levels were measured via tail bleed at baseline and 15, 30, 60, 90, and 120 min post-glucose injection using an automated glucometer (One touch Ultra; LifeScan, Inc., Milpitas, CA, USA). Following a 4 h fast, blood was collected via retro-orbital bleed into a BD Microtainer tube with K_2_EDTA. Samples were centrifuged at 8000×*g* for 10 min at 4 °C, and the plasma was analyzed for triglyceride (TG), cholesterol, and glucose concentration using an enzymatic colorimetric assay kit (Infinity™ TG, Cholesterol, and Glucose assays from Thermo Scientific). Plasma insulin was measured using the Mouse Ultrasensitive Insulin Elisa from ALPCO (cat # 80-INSMSU-E10, ALPCO, Salem, NH, USA).

### Liver triglycerides

For the determination of TG content, the homogenates from the liver tissues were extracted using a methanol–chloroform mixture according to the Folch method [22]. The TG content of each sample was measured after evaporation of the organic solvent using the TG measurement reagent (Thermo Scientific, NH, USA) according to the manufacturer’s instructions.

### Adipose and liver mRNA quantification

RNA was extracted from homogenized frozen epididymal adipose and liver samples with the Maxwell® 16 LEV simplyRNA Tissue Kit by Promega (cat # AS1280, Promega Corp., WI, USA). Total RNA was reverse transcribed into cDNA with random primers using Applied Biosystems cDNA Synthesis Kit (cat #4368814, ThermoFisher, MA, USA). Quantitative PCR (qPCR) was performed using KAPA Sybr Fast qPCR Mix (cat #kk4610, Roche, CA, USA). Copy numbers were normalized to RPL4. Primers used can be found in Additional file 1: Table S1.

### Statistics

Results are presented as means ± SEM. Genotype by strain interaction effect was assessed using a two-way ANOVA test. Tukey's multiple comparisons, as indicated, was performed to assess individual differences among strains and genotypes. Data were analyzed using GraphPad Prism. Significant differences are indicated with symbols defined within the legend of each figure; where no symbol is indicated, differences of mean values did not reach statistical significance (*P* ≥ 0.050).

## Results

### CD44 deficiency reduces diet-induced adiposity

**Fig. 1:**
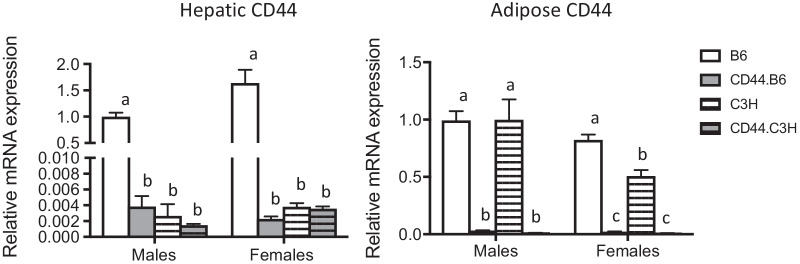
CD44 gene expression. Wild type (B6 and C3H) and CD44-deficient (CD44.B6 and CD44.C3H) mice were fed a high fat, high sucrose, high cholesterol diet for 12 weeks. mRNA was isolated from the liver (**A**) and epididymal white adipose tissue (**B**) to detect CD44 gene expression. mRNA levels were normalized to B6 males. Data are mean ± SE (*n* = 12–15). Bars labeled with different letters are *P* < 0.01 by ANOVA

We hypothesized that the relationship between CD44’s and diabetes may only be evident in an obese state or perhaps in genetically susceptible individuals. In order to test this hypothesis, we used an obesity-susceptible strain of mice, C57BL/6J (B6), and an obesity-resistant strain, C3H/HeJ (C3H) with and without an inactivated, “knocked-out”, CD44 gene (CD44-KO). To generate C3H/HeJ with an inactive CD44 gene, CD44-deficient B6 (CD44.B6) mice were selectively backcrossed into C3H mice to create CD44.C3H mice for 10 generations using marker-assisted breeding. We first assessed the expression of CD44 in both adipose and hepatic tissue and noted a significant strain-dependent expression profile where CD44 expression is significantly reduced (~ 100-fold) in the liver of C3H mice but not adipose tissue (Fig. 1). Notably, the hepatic expression of CD44 in C3H mice is similar in magnitude to that in CD44.B6 and CD44.C3H mice.

**Fig. 2:**
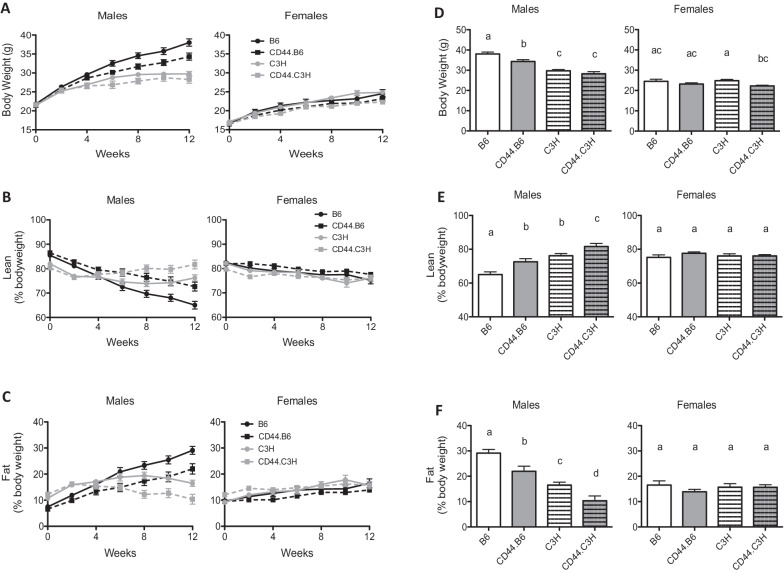
Body weight and adiposity are reduced in CD44-deficient male mice. Wild type (B6 and C3H) and CD44-deficient (CD44.B6 and CD44.C3H) mice were fed a high fat, high sucrose, high cholesterol diet for 12 weeks. **A** to **C** Body weight and percent lean and fat mass were measured throughout diet treatment. Final body weight (**D**), percent lean mass of body weight (**E**), and percent fat mass of body weight (**F**). Data are mean ± SE (*n* = 12–15). Bars labeled with different letters are *P* < 0.05 by ANOVA

All four groups were fed a high fat, high sucrose, high cholesterol diet for 12 weeks to stimulate diet-induced obesity. Over the course of the 12-week diet treatment, all groups gained weight; however, male CD44.B6 gained significantly less weight compared to B6 mice. The total weight gain in B6 males was 16.2 ± 0.8 g, while it was 12.9 ± 0.9 g in CD44.B6 males (Fig. 2A and Additional file 2: Fig. S1A). Thus, male CD44.B6 mice had a 10% reduction in total body weight compared to male B6 mice (*P* < 0.01). This translates to a 20.5% reduction in body weight gain over 12 weeks in CD44.B6 male mice compared to B6 males (*P* < 0.01) (Additional file 2: Fig. S1A). While the final body weight was not lower in CD44.C3H males compared to C3H males, both C3H and CD44.C3H males gained approximately 8.2 g less weight than B6 male mice (Fig. 2A, D). In fact, C3H and CD44.C3H males had a 21.8% and 25.6% lower final body weight compared to B6 males, respectively (Fig. 2A, D). In females, there was no difference in final body weight or weight gain between B6 and CD44.B6 (Fig. 2A, D, and Additional file 2: Fig. S1A). Furthermore, there was no difference among these two groups and C3H females in final body weight or weight gain (Fig. 2A, D, and Additional file 2: Fig. S1A). However, there was a statistically significant reduction (*P* < 0.05) of 10.3% in the final body weight of CD44.C3H females as compared to C3H females (Fig. 2D).

**Fig. 3:**
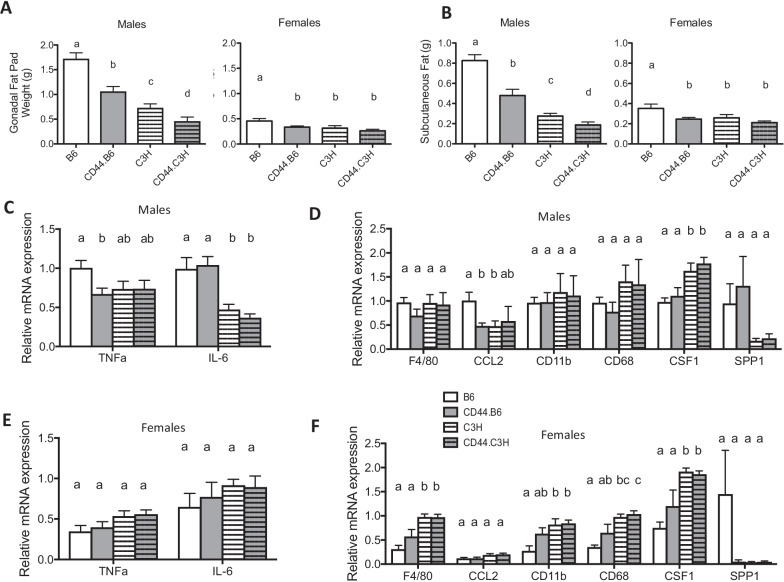
White adipose weight and gene expression. Fat depot weights from gonadal epididymal fat pads (**A**) and subcutaneous inguinal fat (**B**) from wild-type (B6 and C3H) and CD44-deficient (CD44.B6 and CD44.C3H) mice fed a high fat, high sucrose, high cholesterol diet for 12 weeks. mRNA was isolated from epididymal white adipose tissue, and the expression of inflammation-related genes (**C**, **E**) and macrophage-related genes (**D**, **F**) are presented with mRNA levels normalized to B6 male for both males (**C**, **D**) and females (**E**, **F**). Data are mean ± SE (*n* = 12–15). Bars labeled with different letters are *P* < 0.05 by ANOVA

To accurately characterize adiposity development, we measured lean and fat mass accumulation in all mice. Body composition data revealed that in males, lean mass was increased on a C3H background as compared to a B6 background (Fig. 2B, E). Furthermore, CD44 deficiency resulted in leaner mice as compared to their wild-type counterparts. On a B6 background, lean mass percentage increased from 65 ± 1.6% in B6 to 72 ± 1.8% in CD44.B6 male mice (mean ± SEM), and on a C3H background, lean mass increased from 76 ± 1.3% in B6 to 82 ± 1.8% in CD44.C3H male mice (Fig. 2E). In addition to increased lean mass, the inverse results were found in fat mass accumulation in male mice. Male mice on a C3H background had a lower fat mass percentage as compared to male mice on a B6 background (Fig. 2C, F). On a B6 background, fat mass percentage decreased from 29 ± 1.5% in B6 to 22 ± 2.0% in CD44.B6 male mice, and on a C3H background, fat mass decreased from 17 ± 1.2% in B6 to 10 ± 1.9% in CD44. C3H male mice (Fig. 2F). Evidence of reductions in fat mass accumulation was confirmed in white adipose weight measurement in both epididymal and subcutaneous fat (Fig. 3A, B). For example, epididymal fat pads weighed 1.7 ± 0.12 g in B6 males but only 1.1 ± 0.1 g in CD44.B6 male mice (mean ± SEM). On a C3H background, the lean mass decreased from 0.7 ± 0.1 g in C3H males to 0.5 ± 0.1 g in CD44.C3H males (Fig. 3A). Similar results were observed in the subcutaneous fat depots (Fig. 3B). These results indicate that CD44 deficiency in male mice results in leaner mice with less white fat accumulation. These effects were sexually dimorphic, and in female mice, there was no significant change in lean or body fat percentage among all groups (Fig. 2B, C, E, F and Additional file 2: Fig. S1). We also did not observe a statistically significant difference in fat pad weights when CD44 was deleted in female B6 or C3H mice (Fig. 3A, B), indicating CD44 expression did not affect female adiposity in either strain.

### Reduced macrophage marker expression in adipose of CD44-deficient mice

As CD44 is a mediator of cell adhesion in inflammation, we measured inflammatory marker gene expression in adipose tissue from the gonadal epididymal fat pad. Expression of *TNF-α* was significantly reduced 35% in CD44.B6 male mice as compared to male B6 mice (Fig. 3C). Male mice on a C3H background also demonstrated a reduction in *IL-6* and *TNF-α* of 22–25% and 44–63%, respectively, as compared to B6 male mice; however, there was no difference in their expression when CD44 was deleted on the C3H background (Fig. 3C). In both strains (B6 and C3H), expression of *IL-6* and *TNF-α* were found lower in females compared to males (Fig. 3C, E). Furthermore, expression of *CD44* in females of either strain did not affect the expression of the inflammation markers (Fig. 3E). These findings suggest that inflammation markers *IL-6* and *TNF-α* are increased only in the male B6 mice, which had the highest adiposity of all groups.

CD44 has been reported to play a role in macrophage-mediated inflammation [7], so we measured gene expression of macrophage markers *F4/80*, *CCL2, CD11b, CD68, CD14,* and *CSF1* in gonadal epididymal fat tissue. We found that CD44.B6 males had significantly reduced expression of *CCL2,* and a trend for a reduction was demonstrated for *F4/80* and *CD68*, as compared to male B6 mice (Fig. 3C). Interestingly, there were no such reductions observed in male mice on a C3H background as none of the macrophage markers were differentially expressed between C3H and CD44.C3H male mice. Furthermore, there was no significant change in the expression of macrophage markers in females regardless of B6 or C3H background (Fig. 3F). These findings provide evidence that macrophage-associated inflammation in adipose is reduced in CD44.B6 male mice, but not on a C3H background, suggesting that TLR4 signaling is critical to initiating the adipose macrophage system.

### CD44 deficiency improves glucose tolerance in an insulin-resistant model

**Fig. 4:**
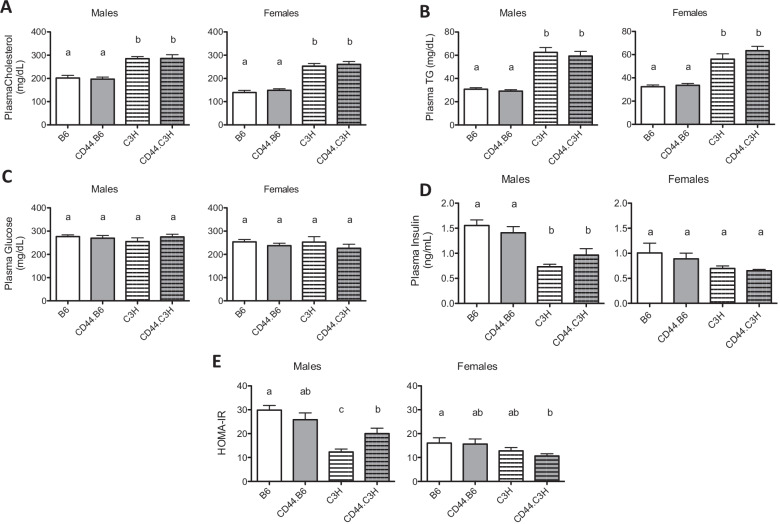
Plasma lipid chemistry is unchanged due to CD44 deficiency. Following 12 weeks on diet, wild-type (B6 and C3H) and CD44-deficient (CD44.B6 and CD44.C3H) mice were euthanized after a 4-h fast, and blood was collected. Plasma cholesterol (**A**), triglycerides (**B**), glucose (**C**), and insulin (**D**) were measured, and HOMA-IR was calculated (**E**). Data are mean ± SE (*n* = 12–15). Bars labeled with different letters are *P* < 0.05 by ANOVA

While adiposity was reduced in CD44-deficient male mice, they did not demonstrate a significant reduction in plasma cholesterol or triglycerides on either strain background for either sex (Fig. 4A, B). Both plasma cholesterol and TG levels were elevated on the C3H background as compared to those on the B6 background in both sexes (Fig. 4A, B. Additionally, there was no change in fasting plasma glucose or insulin levels (Fig. 4C, D) among any of the groups for either sex due to CD44 deficiency. However, male mice on a C3H background had reduced plasma insulin levels when compared with males on the B6 background (Fig. 4D). A similar reduction was seen in the HOMA-IR for mice on the C3H background (Fig. 4E). Interestingly, when HOMA-IR was calculated, a significant increase in HOMA-IR for CD44.C3H mice was detected, as compared to C3H mice (Fig. 4E). There were no significant changes in HOMA-IR between B6 and CD44.B6 mice or among females from either strain.

**Fig. 5:**
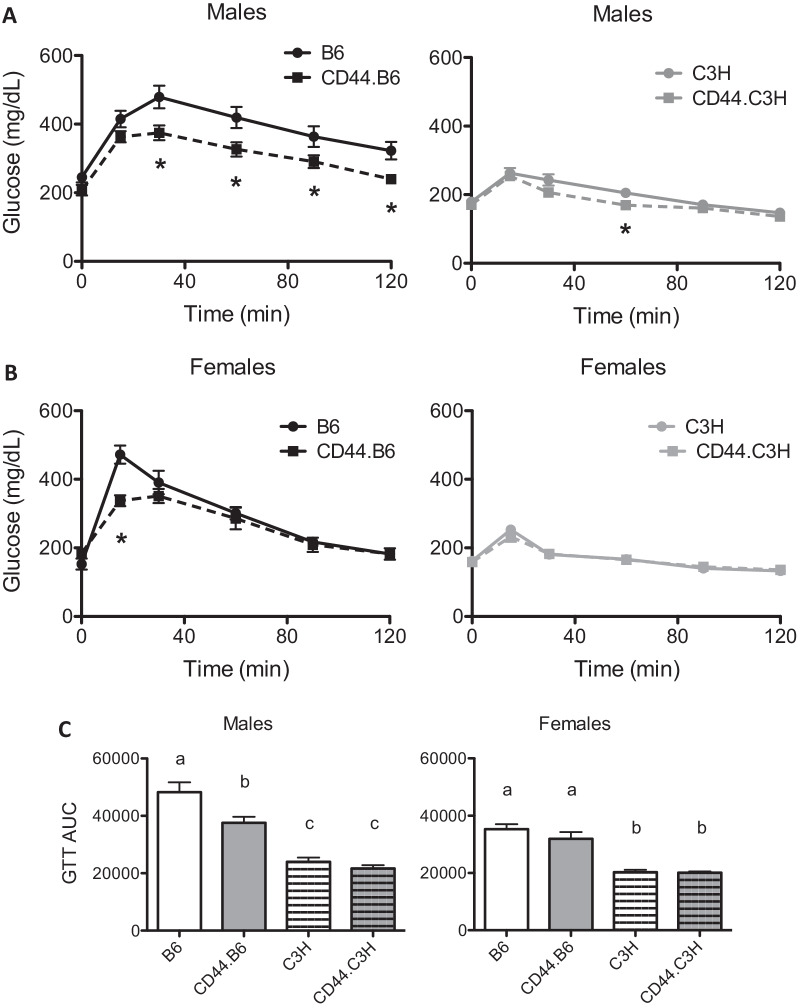
CD44-deficient mice have improved glucose tolerance. Following 10 weeks on diet, wild-type (B6 and C3H) and CD44-deficient (CD44.B6 and CD44.C3H) mice were administered an intraperitoneal injection of 1 g/kg glucose. Using a glucometer, glucose was measured from blood samples collected at 0, 15, 30, 60, 120 min post-glucose injection in males (**A**) and females (**B**), and the area under the curve (AUC) was calculated (**C**). Data are mean ± SE (*n* = 8). Time points labeled with * are *P* < 0.05 by unpaired Student’s t-test. Bar graphs labeled with different letters are *P* < 0.05 by ANOVA

However, when challenged with a glucose tolerance test, CD44.B6 mice demonstrated improved glucose tolerance as compared to B6 mice when challenged with 1 g/kg glucose. Male CD44.B6 mice had improved clearance of glucose from the plasma over 2 h, with a significant reduction in area under the curve of 22%, as compared to B6 male mice (Fig. 5A). Males on a C3H background had a robustly improved glucose clearance compared to B6 mice, with a 50% reduction in area under the curve (Fig. 5A). As glucose tolerance was improved on the C3H background, the effects of CD44 deficiency were less dramatic. In fact, male CD44.C3H mice only had one time point in which the glucose clearance response was significantly reduced when compared to male C3H mice, and a reduction in area under the curve did not reach significance (Fig. 5C). Interestingly, despite no change in adiposity, female CD44.B6 mice did demonstrate an increase in glucose clearance as compared to B6 females (Fig. 5B), although the difference was much more muted than that seen in male B6 mice with reduced plasma glucose at the 15 min time point only. Females on a C3H background had no change in glucose tolerance observed (Fig. 5B, C). These data suggest that ablation of CD44 expression improves glucose tolerance only in male C57BL/6J mice, which are commonly used to study insulin resistance.

### Hepatic lipid accumulation

**Fig. 6:**
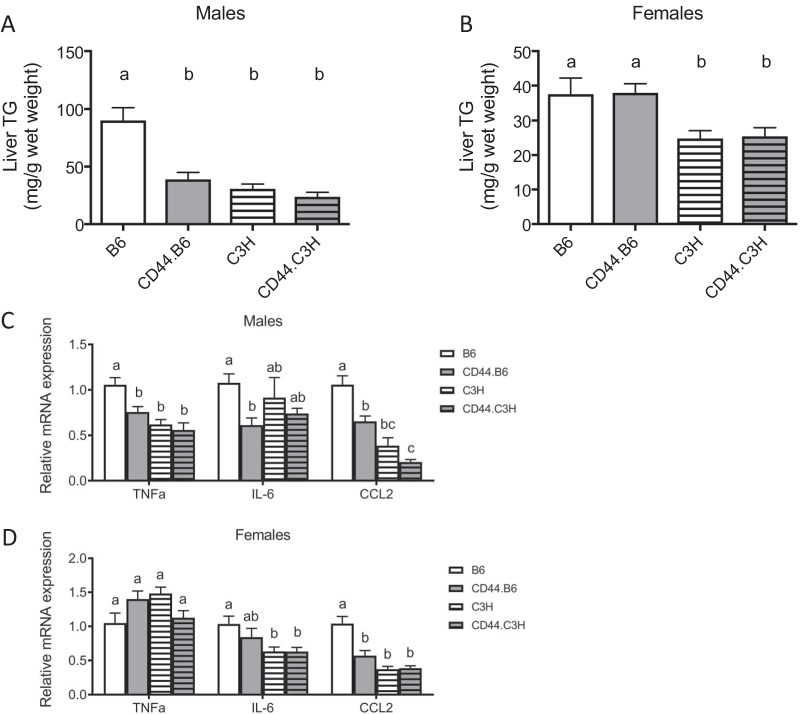
Hepatic lipid accumulation. Following 12 weeks on diet, wild-type (B6 and C3H) and CD44-deficient (CD44.B6 and CD44.C3H) mice were euthanized and liver tissue was collected. Hepatic TG was measured in males (**A**) and females (**B**). Hepatic mRNA was isolated and expression of genes involved in inflammation in males (**C**) and females (**D**) are represented with mRNA levels normalized to B6 mice. Data are mean ± SE (*n* = 12–15). Bars labeled with different letters are *P* < 0.05 by ANOVA

Since obesity and insulin resistance are commonly associated with hepatic steatosis, hepatic TG content was measured in CD44-deficient mice. The TG content of both suggests that, despite 12 weeks of a high fat, high sucrose diet, hepatic steatosis was not induced in C3H or CD44.C3H mice. However, hepatic steatosis was induced in B6 male mice. CD44 deficiency in the B6 background inhibited the development of hepatic steatosis; CD44.B6 mice had a 56.7% reduction in hepatic TG content compared to B6 males (Fig. 6). The TG content of B6 males was increased 2.9-fold and 3.8-fold compared to C3H and CD44.C3H males, respectively. However, this effect was not recapitulated in the female mice, as CD44 deficiency did not reduce hepatic TG levels in either background and hepatic steatosis was not induced in any female group.

### Pro-inflammatory hepatic gene expression is sexually dimorphic by CD44 expression

To determine if the observed decreased adiposity and decreased hepatic lipid accumulation in CD44-deficient mice were driven by hepatic regulation of lipid metabolism, markers of de novo lipogenesis, beta-oxidation, and gluconeogenesis were measured. Hepatic lipogenic genes *FAS, SREBP1, SCD1*, and *ACC1* were unchanged in CD44-deficient mice when compared to their wild-type counterparts for both strain backgrounds in both sexes (Additional file 2: Fig. S2A). However, mice on a C3H background had reduced expression of *SCD1* and *ACC1* compared to B6 mice for both sexes (Additional file 2: Fig. S2A). Similar to lipogenic genes, expression of hepatic beta-oxidative genes *ACC2, ACOX, ACAD*, and *CPT1A* were unaffected by CD44 deficiency on both C3H and B6 backgrounds in both males and females (Additional file 2: Fig. S2B). When comparing strain backgrounds, C3H mice had increased *ACC2* expression and reduced *ACOX* expression as compared to mice on a B6 background (Additional file 2: Fig. S2B). Additionally, expression of gluconeogenic genes *PEPCK, G6P, FBP1*, and *PPARGC1A* were not different with deletion of *CD44* expression in either strain background (Additional file 2: Fig. S2C). However, expression of *PEPCK, G6P*, and *FBP1* were reduced in mice on a C3H background as compared to those on a B6 background in both sexes (Additional file 2: Fig. S2C). These data indicate that *CD44* expression does not modify the expression of hepatic genes for de novo lipogenesis, beta-oxidation, or gluconeogenesis, but mice on a C3H background have reduced expression of gluconeogenic genes and two of the four de novo lipogenic genes measured, as compared to mice on a B6 background.

Given the significant difference in lipid accumulation (Fig. 6A, B) and the subtle effects on gluconeogenic and lipogenic gene expression (Additional file 2: Fig. S2), we next sought to determine if inflammatory pathways were altered in the livers of CD44 mice and if the effect was sexually dimorphic. The expression of *TNFa, IL-6, and CCL2* was reduced by CD44 deficiency in male mice on a B6 background. In females, only CCL2 was significantly reduced 1.8-fold by CD44 inactivation. In both male and female C3H mice, CD44 deletion has minimal effects on the expression of *TNFa*, *IL-6,* or *CCL2* (Fig. 6C, D).

### Metabolic measures in CD44 deficiency

**Fig. 7:**
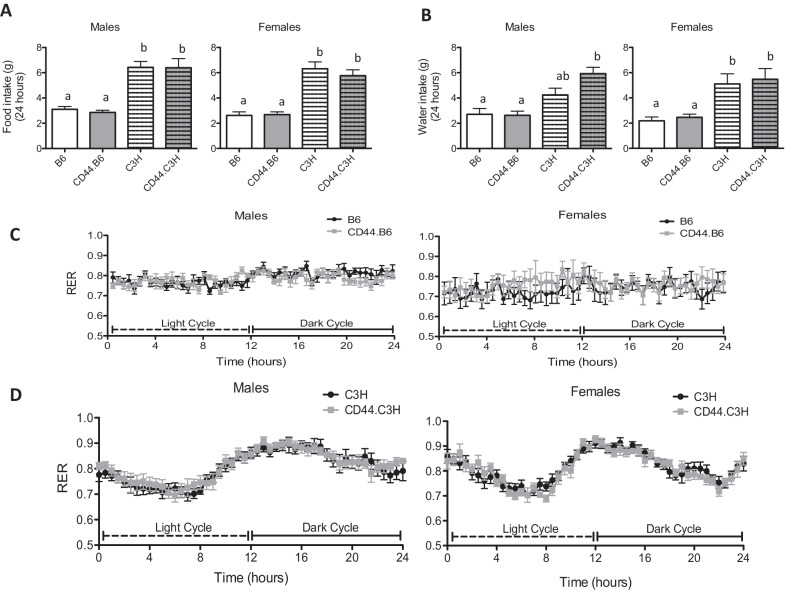
Energy expenditure, and food and water intake were unchanged in CD44-deficient mice. After 11 weeks on diet, wild-type (B6 and C3H) and CD44-deficient (CD44.B6 and CD44.C3H) mice were housed individually in TSE metabolic cages. Food intake (**A**) and water intake (**B**) were measured over 24 h. Energy expenditure as represented by the respiratory exchange rate (RER) for mice on B6 background mice (**C**) and C3H background mice (**D**). Data are mean ± SE (*n* = 8). Bars labeled with different letters are *P* < 0.05 by ANOVA

To determine how CD44.B6 mice may achieve reduced adiposity, mice were individually housed in metabolic cages to measure food intake, activity, oxygen consumption, and carbon dioxide production for 24 h. As shown in Fig. 7A and B, for both strain backgrounds, there was no change in food or water intake caused by CD44 deficiency for either sex. There was also no change in the respiratory exchange ratio for either sex or strain background (Fig. 7C and D). Despite having increased adiposity, mice on a B6 background had a 50% reduction in water and food intake, when compared to mice on a C3H background (Fig. 7A and B). These data indicate that reduced adiposity in CD44-deficient mice cannot be explained by large differences in energy expenditure or food intake.

## Discussion

Obesity is a global epidemic [23], and now, more than two-thirds of adults are considered to be overweight or obese [24]. The increase in obesity is associated with an increased risk of many diseases, including certain cancers, cardiovascular disease, and type 2 diabetes [25, 26]. Understanding the genetic and/or dietary factors contributing to obesity could reduce the overall economic burden of obesity.

In the present study, we focused on a specific factor associated with obesity and diabetes, CD44, and sought to refine our understanding of the relationship between CD44, obesity, and disease risk using genetic models in mice. In our current study, we identified two key findings. First, complete inactivation of CD44 reduces adiposity in two strains of mice but the effect is sexually dimorphic. Second, effects of CD44 on hepatic steatosis and insulin sensitivity were only observed in male C57BL/6J mice. Both of these points are discussed in detail below.

Previous studies in mice demonstrating the relationship between CD44 and obesity have been compelling and clearly demonstrated that CD44 deficiency reduces adiposity and improves insulin resistance [7, 8]. These studies have been performed in C57BL/6J mice, which are genetically susceptible to obesity [27] and insulin resistance [28]. In the current study, we used two mouse models with different susceptibility to obesity and insulin resistance to determine a potential mechanism of action in which CD44 may contribute to the development of obesity. Our present results confirm these findings, as CD44-deficient mice on both a C3H/HeJ and C57BL/6J background had increased lean mass and reduced fat accumulation.

Previous and current results suggest a modification in adipose inflammatory markers, and the primary role of CD44 thus far has been identified in inflammation response, specifically in leukocyte cell-adhesion and recruitment [29]. Thus, we had hypothesized that CD44-deficient mice with perturbed inflammatory response, such as C3H/HeJ mice, would fail to protect the mice from diet-induced obesity. However, the present findings provide evidence that regardless of the susceptibility of the background strain, CD44 deficiency was able to reduce diet-induced obesity in male mice. Male CD44^−/−^ mice on both the C57BL/6J and C3H/HeJ backgrounds were leaner with less fat accumulation than their wild-type counterparts. These effects on adiposity and weight were not observed in female mice of either strain with or without a null CD44 allele. Thus, the effects of CD44 are sex-dependent and specific to the C57BL/6J strain.

However, with respect to adiposity and insulin resistance, the impaired inflammatory response in CD44.C3H mice still allowed for an improvement in obesity development. While C3H mice are more resistant to development of diet-induced obesity [21, 27], CD44 deletion in C3H mice further increased lean mass and reduced fat mass to levels of pre-diet treatment. Thus, despite an impaired inflammatory response, CD44 deletion was still able to improve measures of adiposity, increased lean mass, and reduced adipose mass. Since CD44 was able to reduce adiposity in C3H/HeJ mice, we can infer that the mechanism underlying obesity resistance in CD44-deficient mice is not driven solely by the inflammatory response of the TLR4 pathway.

These data do not suggest the involvement of classic LPS signaling as the mechanism underlying the effects of CD44 deletion on adiposity as C3H/HeJ mice have a functional mutation in the TLR4 gene [20] and have demonstrated an inhibited inflammatory response to LPS. Thus, C3H/HeJ mice serve as a model of impaired inflammatory response. CD44 deficiency in B6 mice reduced adipose inflammatory and macrophage markers, and as expected, CD44 deficiency in C3H mice did not recapitulate a similar decrease. These results are similar to those comparing C3H/HeJ to C3H/HeN mice, which do not have deficient TLR signaling [30]. This is of particular interest as LPS-dependent phenotypes have been studied using macrophages in culture [31] and NASH [32].

The effects of CD44 on glucose tolerance and liver fat accumulation were sexually dimorphic and only evident in C57BL/6J mice. These results confirm previously reported results [32], but also identify one of the more striking results of the current study. The etiology of obesity and subsequent metabolic consequences leading to the development of diabetes is complex. Genetics may underlie the seemingly inconsistent results in our current study. Both C3H and B6 are protected from obesity when CD44 is inactivated, but results varied by mouse strain for liver fat and insulin sensitivity. For example, the diabetic phenotype may be influenced by a recently identified modifier locus on Chromosome 3 in C3H/HeJ mice that is a powerful suppressor of diabetes [33]. The presence of this locus in the C3H and CD44.C3H mice in our study may have influenced the ability to detect improvements in glucose sensitivity in CD44.C3H mice even when body weight and adiposity were improved. Alternatively, these effects may be dependent on hepatic CD44 expression. We observed no endogenous hepatic expression of CD44 in the C3H mice; thus, the muted response to CD44 deficiency in hepatic-driven measures in these mice is understandable. Although there is an underlying mechanism for the difference in susceptibility between C3H and C57BL/6J males, it is important to understand that studies utilizing knockout alleles are context specific, often within the C57BL/6J background. Our results indicate that the underlying susceptibility to glucose intolerance and hepatosteatosis is not caused by CD44 and that the effects of CD44 are only evident in cases of genetic susceptibility. These results highlight one potential reason why translating mouse studies to human therapies is so difficult; most studies have focused on C57BL/6J mice.

We note the dramatic effects of sex in these studies. The effects of a high-fat diet were sexually dimorphic in both strains of mice and independent of CD44 function. This point demonstrates that sex is a critical variable to consider when evaluating disease-relevant candidate genes. Sex effects on obesity and fat distribution are well described in humans [34], and circulating CD44 was demonstrated to be higher in women than men [35]. Studies in mice have also demonstrated sexually dimorphic gene expression patterns in adipose depots [36], and gonadectomy affects gene expression patterns, indicating the profound effects of sex hormones on obesity [37]. Sex chromosome number may also have specific effects on adiposity as well [38].

In conclusion, our studies in mice demonstrated that inactivation of CD44 reduces adiposity in diet-susceptible and diet-resistant mouse strains. The reduced adiposity in CD44.C3H mice indicates that the effects of CD44 on adiposity may be independent of classical TLR signaling, as previously proposed, and further indicates a consistent effect of adiposity. However, metabolic effects on glucose sensitivity and liver adiposity were restricted to C57BL/6J mice. Thus, CD44 remains one of many factors that contribute to the metabolic consequences of calorically rich diets, and additional genetic factors contribute to the development of diabetes and fatty liver disease.

## Supplementary Information

**Additional file 1: Table S1.** Primer sequences for quantitative PCR. **Table S2.** Strain-, genotype-, and strain by genotype effects in traits in each sex. **Table S3.** Strain-, genotype-, and strain by genotype effects in eWAT mRNA expression in each sex. **Table S4.** Strain-, genotype-, and strain by genotype effects in hepatic mRNA expression in each sex.

**Additional file 2: Figure S1. **Changes in body weight. Wild type (B6 and C3H) and CD44-deficient (CD44.B6 and CD44.C3H) mice were fed a high fat, high sucrose, high cholesterol diet for 12 weeks. A: Changes in body weight (g) were calculated as the difference in final body weight and the initial body weight. Changes in percent lean mass of body weight (B) and percent fat mass of body weight (C) were calculated by the difference between the percentages calculated at the final and initial timepoint. Data are mean ± SE (*n* = 12–15). Bars labeled with different letters are *P* < 0.05 by ANOVA. **Figure S2.** Hepatic gene expression. Following 12 weeks on diet, wild-type (B6 and C3H) and CD44-deficient (CD44.B6 and CD44.C3H) mice were euthanized and liver was collected. Hepatic mRNA was isolated and expression of genes involved in de novo lipogenesis (A), beta-oxidation (B), and gluconeogenesis (C) are represented with mRNA levels normalized to B6 male. Data are mean ± SE (*n* = 12–15). Bars labeled with different letters are *P* < 0.05 by ANOVA.

## Data Availability

Please contact author for data requests.

## Abbreviations

TNF-α: Tumor necrosis factor-α
TLR4: Toll-like receptor 4
PEPCK: Phosphoenolpyruvate carboxykinase
G6P: Glucose 6-phosphate
FBP1: Fructose-bisphosphatase 1
PPARGC1A: PPARG coactivator 1 alpha
SREBP1: Sterol regulatory element-binding transcription factor 1
SCD1: Stearoyl-coenzyme A desaturase 1
ACC1: Acetyl-coenzyme A carboxylase 1
ACC2: Acetyl-CoA carboxylase 2
ACOX: Acyl-CoA oxidase 1
CPT1A: Carnitine palmitoyltransferase 1A

## Declarations

### Disclaimers

The USDA is an equal opportunity employer.
