# Supplementary material for: Obesogenic and diabetic effects of CD44 in mice are sexually dimorphic and dependent on genetic background
Source: Biol Sex Differ. 2022 Apr 11;13:14. doi: 10.1186/s13293-022-00426-2 (PMC8996418; doi:10.1186/s13293-022-00426-2)
Supplement: Supplementary file 2 — Additional file 2: Figure S1. Changes in body weight. Wild type (B6 and C3H) and CD44-deficient (CD44.B6 and CD44.C3H) mice were fed a high fat, high sucrose, high cholesterol diet for 12 weeks. A: Changes in body weight (g) were calculated as the difference in final body weight and the initial body weight. Changes in percent lean mass of body weight (B) and percent fat mass of body weight (C) were calculated by the difference between the percentages calculated at the final and initial timepoint. Data are mean ± SE (n = 12–15). Bars labeled with different letters are P < 0.05 by ANOVA. Figure S2. Hepatic gene expression. Following 12 weeks on diet, wild-type (B6 and C3H) and CD44-deficient (CD44.B6 and CD44.C3H) mice were euthanized and liver was collected. Hepatic mRNA was isolated and expression of genes involved in de novo lipogenesis (A), beta-oxidation (B), and gluconeogenesis (C) are represented with mRNA levels normalized to B6 male. Data are mean ± SE (n = 12–15). Bars labeled with different letters are P < 0.05 by ANOVA. [file 13293_2022_426_MOESM2_ESM.docx]

A

B

C

**Figure S1. Changes in body weight.** Wild type (B6 and C3H) and CD44 deficient (CD44.B6 and CD44.C3H) mice were fed a high fat, high sucrose, high cholesterol diet for 12 weeks. **A:** Changes in body weight (g) were calculated as the difference in final body weight and the initial body weight. Changes in percent lean mass of body weight (**B**) and percent fat mass of body weight (**C**) were calculated by the difference between the percentages calculated at the final and initial timepoint. Data are mean ± SE (n=12-15). Bars labeled with different letters are P<0.05 by ANOVA.


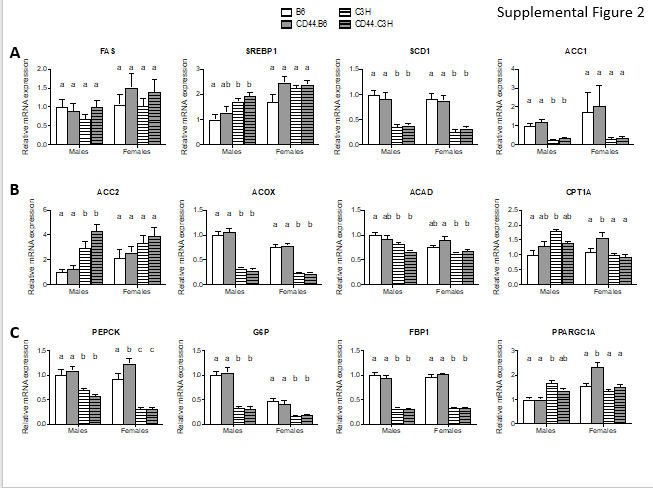


**Figure S2. Hepatic gene expression.** Following 12 weeks on diet, wild type (B6 and C3H) and CD44 deficient (CD44.B6 and CD44.C3H) mice were euthanized and liver was collected. Hepatic mRNA was isolated and expression of genes involved in de novo lipogenesis (**A**), beta-oxidation (**B**), and gluconeogenesis (**C**) are represented with mRNA levels normalized to B6 male. Data are mean ± SE (n=12-15). Bars labeled with different letters are P<0.05 by ANOVA.
